# Cytogenetic abnormalities in essential thrombocythemia: Clinical and molecular correlates and prognostic relevance in 809 informative cases

**DOI:** 10.1038/s41408-022-00639-z

**Published:** 2022-03-17

**Authors:** Naseema Gangat, Yamna Jadoon, Natasha Szuber, Curtis A. Hanson, Alexandra P. Wolanskyj-Spinner, Rhett P. Ketterling, Animesh Pardanani, Ayalew Tefferi

**Affiliations:** 1 Division of Hematology, Mayo Clinic, Rochester, MN USA; 2 Department of Hematology, Université de Montréal, Montréal, QC Canada; 3 Division of Hematopathology, Mayo Clinic, Rochester, MN USA; 4 Division of Laboratory Medicine and Cytogenetics, Mayo Clinic, Rochester, MN USA

**Keywords:** Myeloproliferative disease, Myeloproliferative disease

## Abstract

Cytogenetic studies among 809 consecutive patients with essential thrombocythemia (ET; median age 59 years; 65% females) revealed normal karyotype in 754 (93%), loss of chromosome Y only (-Y) in 16 (2%), and abnormalities other than -Y in 39 (4.8%), the most frequent being sole 20q- (*n* = 8). At presentation, abnormal karyotype, excluding -Y, was associated with older age (*p* = 0.04), higher leukocyte count (*p* = 0.03) and arterial thrombosis history (*p* = 0.02); no associations were apparent for *JAK2/CALR/MPL* mutations whereas *ASXL1* mutations clustered with normal karyotype/-Y and *TP53* with abnormal karyotype. Survival was significantly shorter in patients with abnormal karyotype or -Y, compared to those with normal karyotype (median 12, 10, and 21 years, respectively; *p* < 0.0001). During multivariable analysis that included IPSET (international prognostic score for ET) variables, abnormal karyotype (*p* < 0.01, HR 2.0), age >60 years (*p* < 0.01, HR 4.5), leukocytosis >11 × 10^9^/L (*p* < 0.01, HR 1.5), and male gender (*p* < 0.01, HR 1.4) were independently associated with inferior survival; abnormal karyotype and age >60 years remained significant, along with *SF3B1/SRSF2/U2AF1/TP53* mutations (*p* = 0.04; HR 2.9), when the latter was included in the multivariable model. The current study suggests prognostic relevance for karyotype in ET.

## Introduction

Cytogenetic abnormalities are relatively rare in the context of essential thrombocythemia (ET) with incidence of <10% [1, 2], and unlike the case with polycythemia vera (PV) and primary myelofibrosis (PMF), the prognostic relevance of such abnormalities remain ill-defined [3, 4]. In a Mayo clinic study of 1,076 patients with ET followed for a median of 20 years, overall survival was 37, 22–11 years for patients < 40 years, 41–60 years and > 60 years [5, 6], with reported fibrotic and leukemic transformation rates of 13 and 2.6%, respectively [5]. Conventional survival assessment in ET is based on the International Prognostic Score for ET (IPSET‐survival) which includes age > 60 years, leukocytosis > 11 × 10^9^/L, and prior thrombosis [7], with recent incorporation of spliceosome (*SRSF2/SF3B1*) mutations, age > 60 years and male gender, in the mutation-enhanced international prognostic scoring system (MIPSS-ET) [8]. In that particular study, predictors of disease progression included *U2AF1/SF3B1* mutations for fibrotic and *TP53* mutations for leukemic transformation. Contrary to ET, prognostication in PV (MIPSS-PV) and PMF (MIPSS-70+ version 2.0) relies on abnormal karyotype [8, 9]. Accordingly, in the current study, we utilized a large cohort of 809 consecutive patients with ET fully annotated for karyotype, to describe the prevalence and spectrum of cytogenetic abnormalities, and clinical and molecular correlations of abnormal karyotype in relation to normal karyotype. Importantly, we attempt to discern the clinical implications of such abnormalities in terms of disease evolution and survival in the context of existing prognostic models (IPSET-survival and MIPSS-ET).

## Methods

Patients with ET that fulfilled the World health Organization 2016 diagnostic criteria [10] and underwent evaluation between 1974 and 2021 were retrospectively recruited from our clinical myeloproliferative neoplasm database based on availability of cytogenetic assessment at or within a year of ET diagnosis following Institutional review board (IRB) approval. In order to minimize the inadvertent inclusion of patients with masked PV [11], *JAK2* mutated cases with hemoglobin (Hb) level >16 g/dL in women and 16.5 g/dL in men were excluded from our ET cohort (*n* = 22); similarly, cases with anemia defined by sex adjusted Hb level of <11 g/dL in women (*n* = 39) and <12.5 g/dL in men (*n* = 40) were also excluded, in order to avoid inadvertent inclusion of patients with prefibrotic MF [12]. Moreover, an alternative explanation for anemia i.e., hemoglobin below the reference range of 11.6 g/dl and 13.2 g/dl in females and male patients, respectively was identified in 48 patients, which included gastrointestinal blood loss (*n* = 40), post-operative bleeding (*n* = 2), recurrent epistaxis (*n* = 1), central nervous system bleed (*n* = 1), end stage renal disease (*n* = 3), sickle cell disease (*n* = 1). Analysis considered variables obtained at time of diagnosis. Comparison between categorical variables was performed by Chi square test and continuous variables by Wilcoxon/Kruskal–Wallis tests. Cox proportional hazards model was used to compute multivariable analyses. *P*-value ≤ 0.05 was considered significant. JMP Pro 16.0.0 software package, SAS Institute, Cary, NC was utilized for all analyses.

## Results

809 of 1045 patients had cytogenetic studies performed either at *(n* = 595*)* or within one year *(n* = 214*)* of diagnosis based on timing of referral and reported according to the 2021 International System for Human Cytogenetic Nomenclature [13]. Next generation sequencing-based mutational screening for myeloid relevant genes was performed for research purposes in a subset of cases (*n* = 224) using archived DNA from the first bone marrow assessment (at or within one year of diagnosis). Disease status and survival information was updated in May 2021. All categorical variables are summarized as frequency (percentage), and continuous variables as median (range). Comparison between categorical variables was performed by Chi square test and continuous variables by Wilcoxon/Kruskal–Wallis tests. Cox proportional hazards model was utilized for multivariable analyses, in order to determine the impact of abnormal karyotype on overall, leukemia-free, myelofibrosis-free, and thrombosis-free survival. A comparison of overall survival amongst patients with or without abnormal karyotype was computed by the Kaplan–Meir method with differences evaluated by the log-rank test. *P*-value ≤ 0.05 was considered significant. JMP Pro 16.0.0 software package, SAS Institute, Cary, NC was utilized for all analyses.

A total of 809 ET patients (65% females, median age 59 years) were evaluated, of which karyotype was normal in 754 patients (93%), abnormal in 55 patients (7%) with loss of Y chromosome (-Y) in 16 (2%), and abnormalities other than -Y in 39 (4.8%) patients. Sole abnormalities other than -Y were noted in 30 (4%) cases with two abnormalities in 8 (1%) and three or more abnormalities identified in 1 (0.1%) patient. The most frequent sole abnormalities included del(20q) (*n* = 8), trisomy 8 (*n* = 2), trisomy 9 (*n* = 2), and del(3p) (*n* = 2). We refer the readers to Table 1 for details regarding each specific cytogenetic abnormality.

**Table 1:** Description of 39 cytogenetic abnormalities among 809 patients with Essential thrombocythemia (ET).

*Sole abnormality*
Deletion 20q
46,XX,del(20)(q11.2q13.3)[18]/46,XX[2]
46,XY,del(20)(q13.1q13.3)[3]/46,XY[17]
46,XY,del(20)(q13.1)[20]
46,XY,del(20)(q11.2q13.1)[20]
46,XY,del(20)(q11.2q13.1)[6]/46,XY[9]
46,XY,del(20)(q11.2q13.1)[11]/46,XY[9]
46,XY,del(20)(q13.1)[20]
46, XX,del (20) (q11.2q13.3)[19]/45, XX, -19[1]
Trisomy 8
46,X,-X,+8[6]/46,XX[14]
47,XX,+8[12]/46,XX[8]
Trisomy 9
47,XX,+9[2]/46,XX[18]
47,XX,+9[2]/46,XX[27]
Deletion 3p
46,XY,del(3)(p1321)[18]/46,XY[2]
46,XX,del(3)(p11p14)[20]
Other abnormalities
46,XX,del(5)(q15q33)[10]/46,XX[10]
46,XX,del(5)(q13q33)[2]/46,XX[29]
46,XY,t(4;6)(q23;p11.2)[20]
46,XX,t(2;17)(q37;q21)[16]/46,XX[4]
45,X,-X[10]/46,XX[20]
46,XY,del(13) (q12q14)[13]/46,XY[7]
46,XY,add(21)(p12)[2]/46,XY[18]
46,XX,del(7)(q22)[29]/46,XX[1]
46,XX,del(16)(q22q24)[6]/46,XX[14]
46,XX,add(3)(q21)[4]/46,XX[18]
45,XX,der(14;18)(q10;q10)[12]/46,XX[8]
46,XX,t(11;20)(q?13;q?13)[4]/46,XX[16]
46,X,inv(X)(p11.2q28)[20]/46,XX[6]
46,XX,t(3;11)(p25;q13)[2]/46,XX[18]
46,XY,t(6;12)(q25;q15)[20]
46,XY,t(4;22)(q21;q13)[2]/46,XY[28]
*Two abnormalities*
47,XX+9 [1]/48,XX,+8,+9 [2]/46,XX[28]
48,XX,+8,+9[1]/47,XX,+8[3]/47.XX,+9[11]/46,XX[5]
48,XY,+Y,+9[6]/47,XY,+Y[5]/48,XY[14]
46,XY,add(18)(p11.2)[5]/46,XY,del(20)(q11.2q13.1)[2]/46,XY[13]
46,XX,del(5)(q33),del(11)(q21q25)[12]/46,XX[8]
46,XX,+2mar[5]/92,XXXX,+2marx2/46,XX[13]
46,XY,der(7)t(7;?)(p11.2;?),t(13;20)(q14;q13.1)[3]/46,XY[18]
47,XX,der(7)t(1;7)(q12;p22),+9[3]/46,XX[17]
*Three or more abnormalities*
59,XX,+X,+1,-2,+5,+6,+7,+9,+11,+11,+12,+13,+14,+14,-15,+17,+19,+22[1]59,XX,+X,-2,+4,+4,+5,+6,+8,+9,+11,+14,+17,+18,+19,+21,+21[1]/46,XX[28]

**Table 2:** Clinical and laboratory characteristics of 809 patients with Essential Thrombocythemia (ET), stratified by cytogenetic abnormalities.

Variables	All patients *n* *=* 809	Normal Karyotype *n* *=* 754	Abnormal Karyotype *n* *=* 55 *P*-value^¥=^	Abnormal Karyotype excluding -Y *n* *=* 39 *P*-value^¥=^	-Y chromosome *n* *=* 16 *P*-value^¥=^
Age in years, median (range)	59 (18–96)	58 (18–96)	66 (26–91) **0.0003**	64 (26–85) **0.04**	72 (54–91) **0.001**
Age > 60 years, *n* (%)	368 (45)	334 (44)	34 (62)	18 (46)	13 (81)
			**0.01**	0.24	**0.003**
Gender (male), *n* (%)	282 (35)	251 (33)	31 (56)	15 (38)	16 (100)
			**0.0005**	0.50	
Hemoglobin, g/dl, median (range)	13.8 (11–16.4)	13.8 (11–16.3)	14 (11.2–16.4)	13.6 (11.2–16.4)	14.9 (12.6–16.3)
Reference range			0.10	0.96	**0.001**
Males 13.2–16.6 g/dl					
Females-11.6–15 g/dl					
Leukocytes × 10^9^/L, median (range)					
Reference range:	8.5 (3.5–28.1)	8.4 (3.5–28.1)	9.4 (5.2–18.5)	9.4 (5.2–18.5)	9.4 (6.4–18)
3.5–9.6 × 10^9^/L			**0.01**	**0.03**	0.11
Leukocytes ≥ 11 × 10^9^/L, *n* (%)	169 (21)	155 (21)	14 (25)	10 (26)	4 (25)
			0.41	0.46	0.68
Platelets × 10^9^/L, median (range)					
Reference range	840 (356–3470)	843 (356–3470)	810 (469–1921)	772 (469–1921)	832 (497–1500)
157–371 × 10^9^/L			0.44	0.44	0.81
Platelets ≥ 1500 × 10^9^/L, *n* (%)	60 (7)	55 (7)	5 (9)	4 (11)	1 (6)
			0.62	0.49	0.87
Palpable splenomegaly, *n* (%)	109 (14)	99 (13)	10 (18)	8 (21)	2 (13)
			0.29	0.19	0.93
Thrombosis at or prior to diagnosis, *n* (%)	164^a^ (20)	148^a^ (20)	16 (29)/0.10	12 (31)/0.10	4 (25)/0.60
Arterial, *n* (%)	111 (14)	97 (13)	14 (25)/**0.009**	10 (26)/**0.02**	4 (25)/0.15
Venous, *n* (%)	67 (8)	65 (9)	2 (3)/0.19	2 (5)/0.44	0 (0)/0.22
Driver mutations					
*N*, evaluable	*n* *=* 596	*n* *=* 561	*n* *=* 35	*n* *=* 21	*n* *=* 14
*CALR*, *n* (%)	152 (25)	147 (26)	4 (11)	2 (10)	2 (14)
*JAK2*, *n* (%)	368 (62)	339 (60)	29 (83)	19 (90)	10 (71)
*MPL*, *n* (%)	18 (3)	18 (3)	0 (0)	0 (0)	0 (0)
Triple negative, *n* (%)	59 (10)	57 (10)	2 (5)	0 (0)	2 (14)
			0.06	**0.05**	0.64
Next generation sequencing					
*N*, evaluable	*n* *=* 224	*n* *=* 211	*n* *=* 13	*n* *=* 9	*n* *=* 4
*ASXL1*, *n* (%)	10 (4)	8 (4)	2 (15)/**0.05**	0 (0)/0.55	2 (50)/**<0.0001**
*DNMT3A*, *n* (%)	14 (6)	13 (6)	1 (8)/0.82	1 (11)/0.55	0 (0)/0.61
*SF3B1*, *n* (%)	6 (3)	6 (3)	0 (0)/0.54	0 (0)/0.61	0 (0)/0.73
*SRSF2*, *n* (%)	7 (3)	6 (3)	1 (8)/0.33	1 (11)/0.17	0 (0)/0.73
*TET2, n* (%)	22 (10)	21 (10)	1 (8)/0.28	1 (11)/0.91	0 (0)/0.77
*TP53*, *n* (%)	4 (2)	3 (1)	1 (8)/0.10	1 (11)/**0.03**	0 (0)/0.81
*U2AF1*, *n* (%)	2 (1)	2 (1)	0 (0)/0.72	0 (0)/0.76	0 (0)/0.84
Thrombosis after diagnosis, *n* (%)	170^a^ (21)	156^a^ (21)	14^a^ (25)/0.40	9^a^ (23)/0.72	5 (31)/0.30
Arterial, *n* (%)	139 (17)	127 (17)	12 (22)/0.35	8 (21)/0.55	4 (25)/0.39
Venous, *n* (%)	61 (8)	58 (8)	3 (5)/0.54	2 (5)/0.55	1 (6)/0.83
Hemorrhage after diagnosis, *n* (%)	*n* *=* 755	*n* *=* 704	*n* *=* 51	*n* *=* 35	*n* *=* 16
	85 (11)	81 (12)	4 (8)/0.42	3 (9)/0.59	1 (6)/0.51
Transformed to MF, *n* (%)	95 (12)	89 (12)	6 (11)/0.84	5 (13)/0.85	1 (6)/0.49
Transformed to AML, *n* (%)	24 (3)	22 (3)	2 (4)/0.76	2 (5)/0.43	0 (0)/0.49

*MF* Myelofibrosis, *AML* Acute myeloid leukemia.

^a^Patients with both arterial and venous thrombosis; ¥ = *P* value comparing patients with normal vs abnormal karyotype.

Bold values identify statistical significance (*p* < 0.05)

Table 2 provides a comparative analysis of presenting clinical, laboratory, and molecular features, followed by outcomes in regard to thrombosis, myelofibrosis, and leukemic transformation for our cohort of ET patients, stratified by normal vs abnormal vs -Y karyotype. Abnormal karyotype, other than -Y, in comparison with normal karyotype was associated with older age (median age; 64 vs 58 years, *p* = 0.04), higher median leukocyte count (9.4 vs 8.4 × 10^9^/L, *p* = 0.03) and a higher incidence of arterial thrombosis prior to or at diagnosis (26% vs 13%; *p* = 0.02). The latter association of abnormal karyotype with arterial thrombosis was independent of age (*p* = 0.03) but was fully accounted for the higher prevalence of JAK2 mutation (*p* = 0.22). 596 patients were annotated for driver mutations; abnormal/normal/-Y frequencies were 90%/60%/71% for *JAK2*, 10%/26%/14% *CALR*, 0%/3%/0% *MPL* and 0%/10% /14% triple negative (*p* = 0.14). Among 224 informative cases, *ASXL1* mutation was absent in all patients with abnormal karyotype vs 8/211 (4%) with normal karyotype vs 2/4 (50%) with -Y (*p* < 0.0001). Similarly, *SF3B1* mutation was also absent in patients with abnormal karyotype excluding -Y vs 3% in normal karyotype, (*p* = 0.54), whereas *SRSF2* mutation was present in 8 and 3% with abnormal excluding -Y vs normal karyotype, respectively (*p* = 0.33). On the other hand, *TP53* mutation clustered with abnormal karyotype excluding -Y; 11% vs 1% in normal karyotype (*p* = 0.03). Additional phenotypic and molecular associations of sole abnormalities other than -Y, recurrent sole abnormality, del(20q) and two abnormalities in relation to normal karyotype are presented in Table 3. Patients with sole abnormalities other than -Y were older (median age; 65 years vs 58 years; *p* = 0.001), with higher median leukocyte count (9.3 vs 8.4 × 10^9^/L, *p* = 0.03) and demonstrated an age-independent association with history of arterial thrombosis (*p* = 0.007) due to higher prevalence of *JAK2* mutation. Moreover, presence of sole del(20q) depicted a male preponderance (75% vs 33%, *p* = 0.01) and a higher incidence of prior arterial events (38% vs 13%; *p* = 0.04) which was accounted for by male gender (*p* = 0.11).

**Table 3:** Phenotypic and molecular correlations of Essential Thrombocythemia (ET) patients with sole abnormalities, del(20q) and two abnormalities in comparison to normal karyotype.

Variables	Normal Karyotype *n* *=* 754	Sole abnormalities excluding -Y *n* *=* 30 *P*-value	Sole Del (20q) *n* *=* 8 *P*-value	Two abnormalities *n* *=* 8 *P*-value
Age in years, median (range)	58 (18–96)	65 (28–85)	56 (36–76)	50 (26–71)
		**0.001**	0.72	0.22
Age > 60 years, *n* (%)	334 (44)	18 (60)	3 (38)	2 (25)
		0.09	0.70	0.27
Gender (male), *n* (%)	251 (33)	13 (43)	6 (75)	2 (25)
		0.25	**0.01**	0.62
Hemoglobin, g/dl, median (range)	13.8 (11–16.3)	13.6 (11.2–16.4)	13.6 (12.6–16.3)	13.6 (12.4–15.9)
Reference range Males 13.2–16.6 g/dl Females-11.6–15 g/dl		0.96	0.79	0.99
Leukocytes × 10^9^/L, median (range)	8.4 (3.5–28.1)	9.3 (5.2–18.5)	9.5 (6.3–18.5)	9.9 (7.5–11.2)
Reference range: 3.5–9.6 × 10^9^/L		**0.03**	0.05	0.67
Leukocytes ≥ 11 × 10^9^/L, *n* (%)	155 (21)	9 (30)	3 (38)	1 (13)
		0.22	0.25	0.57
Platelets × 10^9^/L, median (range)	843 (356–3470)	798 (469–1921)	657 (469–1921)	679 (518–1582)
Reference range: 157–371 × 10^9^/L		0.62	0.63	0.61
Platelets ≥ 1500 × 10^9^/L, *n* (%)	55 (7)	3 (10)	1 (13)	2 (17)
		0.58	0.58	0.57
Palpable splenomegaly, *n* (%)	99 (13)	5 (17)	2 (25)	3 (38)
		0.59	0.33	**0.05**
Thrombosis at or prior to diagnosis, *n* (%)	148 (20)	10 (33)/0.07	4 (50)/**0.03**	2 (25)/0.71
Arterial, *n* (%)	97 (13)	9 (30)/**0.007**	3 (38)/**0.04**	1 (13)**/**0.97
Venous, *n* (%)	65 (9)	1 (3)/0.31	1 (13)/0.70	1 (13)/0.70
Driver mutations				
*N*, evaluable	*n* *=* 561	*n* *=* 18	*n* *=* 6	*n* *=* 3
* CALR*, *n* (%)	147 (26)	1 (6)	1 (17)	1 (33)
* JAK2*, *n* (%)	339 (60)	17 (94)	5 (83)	2 (67)
* MPL*, *n* (%)	18 (3)	0 (0)	0 (0)	0 (0)
Triple negative, *n* (%)	57 (10)	0 (0)	0 (0)	0 (0)
		**0.04**	0.68	0.92
Next generation sequencing				
N, evaluable	*n* *=* 211	*n* *=* 8	*n* *=* 3	*n* *=* 1
*ASXL1*, *n* (%)	8 (4)	0 (0)/0.57	0 (0)/0.73	0 (0)/0.84
*DNMT3A*, *n* (%)	13 (6)	0 (0)/0.47	0 (0)/0.65	1 (100)/**0.0002**
*SF3B1*, *n* (%)	6 (3)	0 (0)/0.63	0 (0)/0.77	0 (0)/0.86
*SRSF2*, *n* (%)	6 (3)	1 (13)/0.13	1 (33)/**0.003**	0 (0)/0.86
*TET2, n* (%)	21 (10)	1 (13)/0.89	0 (0)/0.82	0 (0)/0.93
*TP53*, *n* (%)	3 (1)	1 (33)/**0.02**	0 (0)/0.84	0 (0)/0.90
*U2AF1*, *n* (%)	2 (1)	0 (0)/0.78	0 (0)/0.86	0 (0)/0.92
Thrombosis after diagnosis, *n* (%)	156 (21)	6 (20)/0.59	2 (25)/0.76	4 (50)/**0.04**
Arterial, *n* (%)	127 (17)	6 (20)/0.65	2 (29)/0.54	2 (25)/0.54
Venous, *n* (%)	58 (8)	0 (0)/0.11	0 (0)/0.41	2 (25)/0.07
Hemorrhage after diagnosis, *n* (%)	*n* *=* 704	*n* *=* 29	*n* *=* 8	*n* *=* 5
	81 (12)	2 (7)/0.44	2 (25)/0.24	1 (20)/0.55
Transformed to MF, *n* (%)	89 (12)	3 (10)/0.76	2 (25)/0.25	2 (25)/0.25
Transformed to AML, *n* (%)	22 (3)	2 (7)/0.24	2 (25)/**0.0004**	0 (0)/0.62

*MF* Myelofibrosis, *AML* Acute myeloid leukemia.

¥ = *P* value is in comparison with normal karyotype.

Bold values identify statistical significance (*p* < 0.05)

At a median follow-up of 9.6 years (range; 0.01–41.2 years), a total of 95 patients (12%) underwent fibrotic transformation: 5 (13%) with abnormal karyotype, 89 (12%) with normal karyotype and 1 (6%) with -Y (*p* = 0.77). On univariate analysis, predictors of fibrotic progression, age > 60 years (*p* = 0.02), male gender (*p* = 0.04) and *SF3B1/U2AF1* mutations (*p* = 0.001) but not abnormal karyotype (*p* = 0.74) or -Y (*p* = 0.95) (Table 4). Leukemic transformation rates were similar amongst patients with abnormal vs normal vs -Y karyotype with respective frequencies of 5%, 3 and 0% (*p* = 0.71) with sole del(20q) as an independent prognostic factor on age-adjusted multivariable analysis (*p* = 0.01, HR 6.5).

**Table 4:** Impact of abnormal karyotype on overall, fibrosis-free, leukemia-free, and thrombosis-free survival in 809 patients with essential thrombocythemia (ET).

Median follow-up 9.6 years (range; 0.01–41.4 years)	Overall survival		Fibrosis-free survival		Leukemia-free survival		Thrombosis-free survival
	Total events = 288(36%) Abnormal karyotype 23(59%) Normal karyotype 257(34%) -Y 8(50%)		Total events = 95(12%) Abnormal karyotype 6(11%) Normal karyotype 89(12%) -Y 1(6%)		Total events = 24(3%) Abnormal karyotype 2(4%) Normal karyotype 22(3%) -Y 0(0%)		Total events = 170(21%) Abnormal karyotype 9(23%) Normal karyotype 156(21%) -Y 5(31%)
	Univariate *P*-value^a^ HR (95% C.I.)	Multi-variate *P*-value HR (95% C.I.)	Univariate *P*-value HR (95% C.I.)	Multi-variate *P*-value HR (95% C.I.)	Univariate P-value HR (95% C.I.)	Multi-variate *P*-value HR (95% C.I.)	Univariate/multivariate *P*-value HR (95% C.I.)
Abnormal karyotype including -Y (*n* = 55)	**0.0005 2.1** **(1.4–3.0)**	IPSET; **<0.0001 10 (7.1–14.9)** Abnormal karyotype; **0.0006 2.0 (1.4–2.9)**	0.78 1.1 (0.5–2.6)		0.56 1.5 (0.3–6.5)		0.09 1.6 (0.9–2.8)
		Age > 60 yrs; **<0.0001 4.4 (3.4–5.7)** Leukocytosis > 11 × 109/L; **0.0007 1.6 (1.2–2.1)** Male gender; **0.006 1.4(1.1–1.8)** Abnormal karyotype; **0.001 1.9 (1.3–2.7)** Prior thrombosis; 0.12					
		Age > 60 yrs; **<0.0001 4.5(2.7–7.4)** *SF3B1/SRSF2/U2AF1/TP53* mut; **<0.0001 3.9 (2.0–7.4)** Abnormal karyotype; **0.07 2.2 (0.9–5.3)** Male gender**;** 0.09 **1.5 (0.9–2.4)** Leukocytosis > 11 × 109/L; 0.36					
Abnormal karyotype excluding -Y (*n* = 39)	**0.007** **1.9 (1.2–2.9)**	IPSET; **<0.0001 10.3 (7.2–15.0)** Abnormal karyotype; **0.001 2.1 (1.4–3.2)**	0.74 1.2 (0.5–2.9)		0.40 1.8 (0.4–7.9)		0.42 1.3 (0.6–2.6)
		Age > 60 yrs; **<0.0001 4.5 (3.4–5.8)** Leukocytosis > 11 × 109/L; **0.002 1.5 (1.2–2.0)** Male gender; **0.005 1.4(1.1–1.8)** Abnormal karyotype; **0.001 2.0 (1.3–3.1)** Prior thrombosis; 0.13					
		Age > 60 yrs; **<0.0001 4.6 (2.8–7.7)** *SF3B1/SRSF2/U2AF1/TP53* mut; **<0.0001 3.9 (2.0–7.5)** Abnormal karyotype; **0.07 2.7 (0.9–7.8)** Male gender**;** 0.09 1.5 (0.9–2.4) Leukocytosis > 11 × 109/L; 0.62					
Loss of Y chromosome (*n* = 16)	**0.003** **2.8 (1.4–5.8)**	IPSET; **<0.0001 10.3 (7.0–15.4)** -Y; 0.06 1.9 (0.9–3.9)	0.95 1.1 (0.2–7.7)		0.50		**0.04** **2.5 (1.0–6.2)** Age-adjusted 0.21
		Age > 60 yrs; **<0.0001 4.9 (3.8–6.4)** -Y; 0.15 1.7 (0.8–3.4)					
Sole abnormalities excluding -Y (*n* = 30)	**<0.0001** **2.6 (1.6–4.3)**	IPSET; **<0.0001 10.0 (6.9–14.4)** Sole abn; **<0.0001 2.8 (1.7–4.6)**	0.64 1.3 (0.4–4.2)		0.11 3.3 (0.8–14.3)		0.99 0.9 (0.4–2.4)
		Age > 60 yrs; **<0.0001 4.6 (3.5–5.9)** Leukocytosis > 11 × 109/L; **0.004 1.5(1.1–1.9)** Male gender; **0.009 1.4 (1.1–1.8)** Sole Abn; **0.0001 2.5 (1.6–4.2)** Prior thrombosis; 0.27					
Del (20q) (*n* = 8)	0.20 1.8 (0.8–4.1)		0.26 2.2 (0.5–9.1)		**0.01** **6.5 (1.4–29.6)**	Age > 60 yrs; 0.89 Del(20q); **0.01 6.5 (1.4–29.7)**	0.75 1.3 (0.3–5.1)
Two abnormalities (*n* = 8)	0.58 1.3 (0.5–3.5)		0.98 1.0 (0.25–4.1)		0.99		0.07 2.5 (0.9–6.7)

^a^All comparisons were performed with normal karyotype.

Bold values identify statistical significance (*p* < 0.05)

**Fig. 1:**
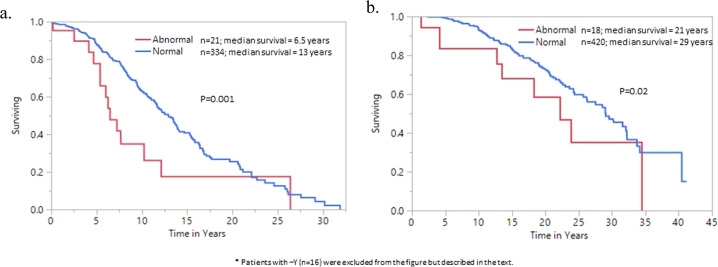
Overall survival and karyotype in essential thrombocythemia. **a** Overall survival of 355 patients of age> 60 years with essential thrombocythemia, stratified by abnormal vs normal karyotype. **b** Overall survival of 438 patients of age < 60 years with essential thrombocythemia, stratified by abnormal vs normal karyotype.

Abnormal karyotype and -Y were both found to be associated with inferior survival with median survival of 12 years (range; 0.1–34) and 10 years (range; 0.01–19.9), respectively, compared to 21 years (range; 0.01–41.2) for normal karyotype (*p* < 0.0001). Figure 1a, b illustrate the adverse impact of abnormal karyotype other than -Y on overall survival in comparison to normal karyotype regardless of age. Moreover, overall survival of patients with loss of Y in > 75% vs < 25% metaphases were significantly shortened at 5 years vs 15 years, (*p* = 0.04), despite similar age, leukocyte count, prior thrombosis history, since limited mutational data was available, further comparison was not performed. However, the survival difference among patients with loss of Y in > 75% metaphases vs abnormal karyotype was fully accounted for by age (median age; 72 years with loss of Y > 75% vs 64 years with abnormal karyotype). In univariate analysis, risk factors for overall survival included abnormal karyotype (*p* = 0.007), -Y (*p* = 0.003), age >60 years (*p* < 0.0001), leukocytosis >11 × 10^9^/L (*p* < 0.0001), male gender (*p* = 0.0003), and history of thrombosis (*p* = 0.001). However, upon multivariable analysis which included IPSET-survival variables, abnormal karyotype other than -Y remained significant (*p* = 0.001, HR 2.0), along with age >60 years (*p* < 0.0001, HR 4.5), leukocytosis >11 × 10^9^/L (*p* = 0.002, HR 1.5), and male gender (*p* = 0.005, HR 1.4) (Table 4). Furthermore, the prognostic impact of abnormal karyotype other than -Y on overall survival remained significant in the presence of *SF3B1/SRSF2/U2AF1/TP53* mutations (*p* = 0.04; HR 2.9).

## Discussion

Amongst the myeloproliferative neoplasms (MPN), cytogenetic abnormalities are relatively infrequent in the context of ET (7%) in comparison to PV (19%) and PMF (45%) (Table 5) [3, 4]. Akin to PV and PMF, the vast majority (76%) were classified as sole abnormalities. Cytogenetic risk stratification has been the cornerstone of prognostic assessment in PMF with incorporation of karyotype in the dynamic international prognostic score (DIPPS plus) [14], and within the contemporary MIPSS70 plus score [9]. Similarly, in PV, the presence of abnormal karyotype has been shown to be detrimental to overall survival, together with an enhanced risk for fibrotic and leukemic progression [3, 8]. Prior investigations in ET have been unable to establish a relationship between abnormal karyotype and overall survival or disease progression, primarily because of the infrequent occurrence of cytogenetic abnormalities [1, 2]. In that regard, the current study is unique since it represents the largest cohort of ET patients fully annotated for karyotype and followed for up to five decades, enabling us to offset the above limitations. As a result, for the first time, we were able to demonstrate an adverse impact of abnormal karyotype other than -Y, on overall survival which was independent of IPSET and adverse mutations. In a prior report on serial cytogenetic analyses in MPN patients, cytogenetic clonal evolution was documented in only 14/153 (9%) patients with ET and frequently associated with clinically overt disease progression in 64% of cases; albeit a change in karyotype from normal to abnormal, without clinical evidence of disease transformation was infrequent, the finding by itself demonstrated a trend for adverse survival in ET [15].

**Table 5:** Comparison of cytogenetic abnormalities in patients with essential thrombocythemia, polycythemia vera, and primary myelofibrosis.

	Essential thrombocythemia (ET)^a^ *n* *=* 809	Polycythemia Vera (PV)^b^ *n* *=* 196	Primary myelofibrosis (PMF)^c^ *n* *=* 1002
Abnormal karyotype, *n* (%)	55 (7)	38 (19)	449 (45)
Sole abnormalities, *n* (%)	46 (6)	34 (17)	320 (32)
Frequent sole abnormalities, *n* (%)			
− −Y	16 (2)	8 (4)	9 (0.9)
− +9	2 (0.2)	9 (5)	14 (1.4)
− +8	2 (0.2)	5 (3)	26 (3)
− del(20q)	8 (1)	5 (3)	74 (7)
− del(13q)			56 (6)
Two abnormalities, *n* (%)	8 (1)	4 (2)	68 (7)
Three or more abnormalities, *n* (%)	1 (0.1)	0 (0)	61 (6)
Favorable karyotype*, *n* (%) Unfavorable karyotype, *n* (%) Very high-risk (VHR) karyotype, *n* (%)		7 (4)	737 (74) 190 (19) 75 (7)
Impact of abnormal karyotype on outcome			
Overall survival	*P* = 0.0005; HR 2.1	*P* = 0.03; HR 1.9	VHR- 1.2 months, HR 3.8 Unfavorable- 2.9 months, HR 1.7 Favorable- 4.4 years *P* < 0.0001
Fibrosis-free survival	*P* = 0.78	*P* = 0.0002; HR 7.8	–
Leukemia-free survival	*P* = 0.56	*P* = 0.004; HR 12.5	VHR- HR 4.4 Unfavorable- HR 2.0 *P* < 0.0001

^a^Current study.

^b^Barraco D et al. [3].

^c^Tefferi A et al. [4].

*PMF- ‘favorable’—normal karyotype or sole abnormalities of 13q−, +9, 20q−, chromosome 1 translocation/duplication or sex chromosome abnormality including -Y; ‘very high risk (VHR)’—single/multiple abnormalities of −7, i(17q), inv(3)/3q21, 12p−/12p11.2, 11q−/11q23, or other autosomal trisomies not including +8/+9 (e.g., +21, +19); ‘unfavorable’—all other abnormalities. PV- Unfavorable karyotype (−7/7q−).

The current study confirms the association of abnormal karyotype in ET with older age, higher leukocyte count, *JAK2* mutation, and its mutual exclusivity with *ASXL1* and *SF3B1* mutations. Additionally, the identification of cytogenetic abnormalities at the time of presentation in a small minority of patients and its association with inferior survival, underscores the value of obtaining cytogenetic studies as part of the diagnostic workup of ET. Notwithstanding the limitations of a retrospective report, every attempt was made to only include informative cases with cytogenetics performed either at or within a year of diagnosis, in order to eliminate the effects of therapies received and inadvertent inclusion of post-ET MF. In regard to the impact of cytoreductive therapies upon disease progression, it remains to be determined if the potential DNA-damaging effect of hydroxyurea might be enhanced in patients with cytogenetic abnormalities and/or additive somatic mutations at the time of diagnosis or when hydroxyurea is instituted. Our observations require clarification from collaborative studies, which should also investigate the effect of specific abnormalities and treatments received.

## Supplementary information

journal checklist
